# The Key Role of Nucleic Acid Vaccines for One Health

**DOI:** 10.3390/v13020258

**Published:** 2021-02-08

**Authors:** Anders Fomsgaard, Margaret A. Liu

**Affiliations:** 1 Department of Virology and Microbiological Special Diagnostic, Statens Serum Institut, 5 Artillerivej, DK-2300 Copenhagen, Denmark; 2 ProTherImmune, 3656 Happy Valley Road, Lafayette, CA 94549, USA

**Keywords:** DNA vaccines, mRNA vaccines, plasmid, immune responses, Cytolytic T Lymphocyte (CTL), one health

## Abstract

The ongoing SARS-CoV-2 pandemic has highlighted both the importance of One Health, i.e., the interactions and transmission of pathogens between animals and humans, and the potential power of gene-based vaccines, specifically nucleic acid vaccines. This review will highlight key aspects of the development of plasmid DNA Nucleic Acid (NA) vaccines, which have been licensed for several veterinary uses, and tested for a number of human diseases, and will explain how an understanding of their immunological and real-world attributes are important for their efficacy, and how they helped pave the way for mRNA vaccines. The review highlights how combining efforts for vaccine development for both animals and humans is crucial for advancing new technologies and for combatting emerging diseases.

## 1. Introduction: Background for Nucleic Acid (NA) Vaccines

It has now been three decades since Felgner and colleagues published that nucleic acid constructs, i.e., both plasmid DNA and mRNA could be directly injected in vivo into mice resulting in the encoded protein made in situ [1]. The potential applications included not simply gene therapy (i.e., provision of correct protein when a gene is missing or mutated) or drug therapy via production of a therapeutic protein, but also vaccines. The challenges for use of nucleic acids as a means of producing therapeutic proteins in vivo included both targeting the correct tissue and having therapeutic levels of the protein made. When an initial study demonstrated that plasmid DNA encoding antigens from a virus, namely, influenza hemagglutinin and nucleoprotein, could result in the generation of immune responses, capable of protecting against homologous and even against a different strain of virus than the one from which the gene for the antigens were taken [2], significant efforts were then directed towards the use of plasmid DNA for vaccines. The DNA vaccines were shown to be capable of generating protective neutralizing antibodies and both helper T cells and Cytolytic T Lymphocytes (CTLS). The T cell help was predominantly of the Th1 phenotype for directly injected DNA (vs. a Th2 phenotype when the plasmid was delivered via coating onto gold beads and propelled biolistically into the skin via a “gene gun”). Additionally, the generation of CTLs achieved a sort of “holy grail” for vaccinologists of being able to consistently generate MHC-Class-I restricted CTL without the use of live virus vaccines [3]. Many immunological mechanisms needed to be demonstrated, including how production of the antigen predominantly by and in muscle cells (and possibly other non-antigen presenting cells) could result in not just antibodies, but also in cytotoxic T lymphocytes (CTL) [4]. The plasmid DNA itself also appeared to contribute by stimulation of the innate system, specifically TLR9, via CpG motifs [5] and possibly other molecules such as AIM2 (Absent In Melanoma 2, a cytolsolic DNA sensor that is part of the innate immune response) [6]. In this regard, DNA vaccines, like mRNA vaccines, are not simply vectors encoding the antigens, but have adjuvant-like activity in and of themselves through stimulation of innate immune responses that likely contribute to the adaptive responses [7]. In addition, safety concerns were raised by injection of genes, including whether or not the plasmid DNA could integrate into chromosomes and whether deleterious immune responses would be generated, either autoimmunity or tolerance. Autoimmunity had been a concern because anti-DNA antibodies are a hallmark of autoimmune diseases so the issue of whether a DNA vaccine could initiate an autoimmune disease was raised. The polar opposite immunological issue of tolerance was raised as a possibility because the low levels of protein/antigen produced by the plasmid DNA by non-professional antigen presenting cells is a mechanism thought to engender tolerance. Because the plasmid DNA transfected muscle cells, which are not professional antigen presenting cells (APCs), and these muscle cells could produce the encoded protein antigen, the question was raised as to whether a DNA vaccine would induce tolerance against an antigen. However, these concerns were put to rest through extensive pre-clinical studies and clinical trials. The results showed that the DNA did not integrate at levels that could be detected above any background mutation rates [8], and that neither autoimmunity nor tolerance were seen [5]. In fact, quite the opposite was seen—strong immune responses against foreign pathogen antigens and tumor antigens could be generated, while therapy of autoimmune diseases such as diabetes [9] and multiple sclerosis [10] looked promising in early phase human clinical trials. These studies paved the way for future NA vaccines, namely mRNA vaccines, as, for example, concerns about autoimmunity and tolerance and potential limits of immune responses due to production of antigen by non-APCs have not been considered crucial.

Significant enthusiasm was raised by pre-clinical and clinical studies showing promising results for using plasmid DNA to prevent or treat diseases as varied as infectious diseases, cancer, autoimmune diseases, and allergy (e.g., asthma) [11]. Further reasons for the enthusiasm included (1) the rapidity and ease of making any plasmid DNA vaccine, since all that is needed is the sequence of the gene encoding the antigen to make the DNA vaccine construct; (2) the ease of manufacturing plasmid DNA; and (3) its stability. In comparison to attenuating a live virus, for example, then finding the right conditions to manufacture it, or even to make a recombinant protein with the correct structure (such as post-translational modifications which depend on the manufacturing host cell), plasmid DNA manufacture has the advantage of being a simple and generic process. Despite a torrent of pre-clinical demonstrations of efficacy of plasmid DNA vaccines in various animal diseases and diverse pre-clinical disease models, and even with promising Phase 1 and Phase 2 clinical trials of DNA vaccines for a number of diseases (reviewed in [5,11] and examples for emerging pathogens described in [12,13,14]), and even with the licensure of various veterinary plasmid DNA vaccines (described below), to date, no human plasmid DNA vaccines have been licensed.

The licensed veterinary plasmid DNA vaccines (as described in the next section) and those certain of the human clinical trials, in diseases as diverse as emerging pathogens to cancer [12,13,14,15,16,17,18], point the way towards potential future successes of DNA vaccines for broader human and veterinary uses. Moreover, what has been learned from the all the studies, whether pre-clinical immunological mechanistic studies or clinical trials, has also paved the way for other types of gene-based vaccines, notably mRNA vaccines, and even for viral vectors including what is being clinically being considered for the current Covid-19 pandemic, namely, heterologous prime-boost immunizations.

This review will describe the advances first in veterinary plasmid DNA vaccines, and will review the relevant aspects of human plasmid DNA vaccines and immunotherapies that have set the stage both for other gene-based vaccines such as mRNA and viral vectors, and for the hoped-for expanded development of plasmid DNA vaccines. All of this will be done in the increasingly important context of One Health, which is the interactions of animal and human health. Understanding the importance of the human and animal ecosystem for the emergence of pathogens and of the immune preventions and therapies for both humans and animals are crucial for anticipating, preventing, and responding to novel pathogens. 

## 2. Veterinary Applications

Within the veterinary field, nucleic acid vaccines have promoted a revolution in the concept of vaccination, which has been previously dominated by inactivated or live vaccines. The improvement of immunogenicity and the easy efficient delivery of NA vaccines provide several rationales for NA vaccination of companion and production animals against classical and emerging veterinary virus diseases, as well as new zoonotic viruses. In general, plasmids seem cost-effective for veterinary use (as exemplified even for fish farming [19,20,21]). The stability of plasmid DNA is high and can be lyophilized, thus eliminating the need for cold chain. The design from known virus sequences can be simple and fast and different genes can be combined simultaneously, making it possible to develop also multivalent vaccines.

The road to obtaining useful veterinary DNA vaccine candidates has been long but with many promising observations (Table 1), and a variety of veterinary NA vaccine candidates have undergone clinical trials over the years [22,23,24,25].The animal diseases most targeted by veterinarian NA vaccines include primarily viral diseases, e.g., foot and mouth disease and herpes virus infection in cattle, Aujeszky’s disease and classical swine fever in swine, rabies and canine distemper in canines, and avian influenza, infectious bronchitis, infectious bursal disease, and coccidiosis in birds [22,23,26,27,28,29] (Table 1). Fish DNA vaccines [19] for infectious hematopoietic necrosis virus was licensed in 2005 [20] and a DNA vaccine against salmon pancreas disease was licensed in 2016 [21], as further discussed below. In addition, promising DNA vaccine candidates against bacterial and protozoan infections, e.g., coccidiosis, were obtained when using the 3-1E and EtMIC2 genes combined with cytokines as adjuvants that protected birds from this economically important infection [30].

In recent years, there seems to be an increased frequency of emerging virus outbreaks among humans. Most of these novel viruses are zoonotic RNA viruses with high mutation potential emerging from wild animals. This is a consequence of increased contact with wild animals and their viruses. This cross-species infection is either directly or after virus amplification and super-spreading events in flocks of humans of flocks of production animals. Examples of this are the 2003 Sars-CoV from bats and mammals [55], the 2009 swine influenza from pigs [56], the 2012 Mers-Cov from bats and camels [55], the 2014 world’s largest Ebola outbreak from bats in Central or West Africa [57], the 2015 Zika virus from mosquitoes and monkeys [58], and the 2019 Sars-CoV-2/Covid-19 with a possible origin from bats [59,60].

The challenge of novel zoonotic virus infections in production animals is the transmission in herds of often thousands of animals with possible spread to many new farms. In addition, several new virus variants can arise by mutations during the spreading through many thousands of individual animals in farms. Such mutations could be adaptation mutations, increased infectivity, and/or pathogenicity in the animals, which may affect also humans, as seen, for example, with influenza [56] and Sars-CoV-2/Covid-19 infections in mink and humans [61]. 

Avian influenza viruses from migrating birds can acquire the multiple basic amino acid mutations in the HA1-HA2 cleavage site causing a highly pathogenic avian influenza virus with high mortality in chickens and other susceptible bird species. In addition, reassortment of influenza virus during infection of swine, bird, and human may cause antigenic shifts creating new influenza viruses with pandemic potential. 

Until 2020, Denmark had about 1300 mink farms each with approximately 10,000–30,000 mink, a number that increases at the time of new puppies. Thus, Denmark was the largest mink producer in Europe with more than 17 million mink concentrated in specific geographic areas in a relatively small country of 5.8 million people. In June 2020, SARS-Cov-2 caused an unstoppable increase in the numbers of infected mink farms in Denmark to more than 288 in the fall of 2020 causing a significant parallel increase in human infections with mink-variant viruses [61]. To improve infection control during the increasing second Covid-19 wave, culling of all mink, infected or not yet infected, was deemed necessary, which did decrease the numbers of human infections by mink variant viruses. Without intensive testing of mink in different countries which have production mink it is difficult to measure the impact of mink-derived SARS-Cov-2 on the epidemic in the different countries. When available, SARS-Cov-2 NA vaccinations of mink may be considered to allow farming of such very susceptible production animals.

Similar concerns may be true during a new human influenza pandemic where exchange of influenza viruses between humans and flocks of production pigs and chickens may develop into multiple uncontrollable virus reservoirs of human concern. A recent example is the triple reassortant H1N1pdm09, composed of genes from three known swine viruses, which was detected in Mexico and the USA. This new virus variant rapidly spread among humans during the 2009 pandemic and was later transmitted from humans into pig populations worldwide [62].

The spillover from infected production animals to humans and back and into wild fauna may contribute to an uncontrollable spread of zoonotic pathogenic viruses and could establish new virus reservoirs among new animal species. Thus, increased and improved broadly reacting NA vaccinations of animals may be useful to protect them from zoonotic viruses and benefit production animal health. This would also protect humans from an occupational risk and reduce the risk to public health. “To protect the humans, vaccinate the animals”.

Zoonotic viruses may cause disease in the animals and/or cause a negative impact on animal production. Swine influenza (SIV) causes reproductive problems, weight loss, and aggravation of secondary infections in pigs, and SIV is a leading infection affecting the majority of herds in modern swine production [63].

Current circulating endemic swine influenza virus strains of, e.g., H1(av)N1, H1N1pdm09, H1(av)N2, H3(hu)N2(sw) may differ slightly from the similar human influenza virus variants [64]. However, broadly protective influenza DNA vaccines have been shown to cross-protect from a variety of drifted influenza stains not included in the DNA vaccine [65], including the highly pathogenic H5N1 avian influenza strains of pandemic potential [66]. Moreover, DNA vaccination of piglets is possible even in the presence of maternal antibodies [67,68] where other vaccines may cause suppression of the immune response and induce vaccine-associated enhanced respiratory disease (VAERD) [69]. Since these two studies were for different pathogens (influenza and pseudorabies virus), this may indicate that this observation may apply to various pathogens, such that DNA vaccines may be given earlier in life to piglets than protein vaccines. 

Since the emergence of the NA vaccination platform in the early 1990s [2], DNA vaccines have shown high and long-lasting protective immunity with few side-effects noted against several different virus infections in several different animal species. This was a vaccine revolution because of the many advantages in using plasmid DNA. Advantages being their easy design from just NA sequence data, high stability of plasmid DNA that could be freeze-dried, the potentially inexpensive production in *Escherichia coli*, and the induction of a long-lasting Th1-biased immune response with broad-reacting antibodies and strong cellular immunity, mimicking live attenuated vaccines without the same biohazards. 

The intracellular production of antigens may explain the efficient induction of protective cell-mediated immunity (CMI) also in primates; however, the lower antibody immunogenicity of plasmid DNA vaccines in primates [70] have slowed down the development to licensed vaccines for human diseases. 

In some cases, DNA vaccines have failed to produce measurable antibodies but with protection of the host which may represent a limitation in measuring protective antibodies or demonstrate a major role for CMI [41,44]. Efforts have therefore been concentrated towards improving the delivery of NA vaccines and increasing their antibody immunogenic potential, particularly since antibody correlates of efficacy have been commonly utilized (and no vaccines have been developed with a cellular correlate of efficacy).

The recent improvement in gene design, optimized codon-change in the vaccine genes and improved plasmid vectors [71,72], or the use of pseudo-uridine in mRNA [73,74], encapsulation of mRNA in nanoparticles [75] or the use of adjuvants [76] and special delivery methods such as gene guns, electroporation and needle-free jet-injection [5] to very immunogenic organs like the skin, have significantly improved nucleic acid vaccines (with the improvements listed here utilized variably for DNA and mRNA vaccines). These improvements of NA vaccine technology and better choice of target diseases have successfully changed the field. These improved genetic vaccines, especially the mRNA vaccines are now able to induce significant immune responses in humans demonstrably protective, as will be discussed below. 

Many veterinary DNA vaccine candidates have been evaluated over the years leading to valuable observations and three licensed vaccines (Table 1). 

Cattle: These include vaccine candidates against viral diseases of bovines where the use of DNA vaccines encoding the VP4 protein of bovine rotavirus (BRV) was first found effective in stimulating a Th1-like immune response [31]. In 1999, Brillowska et al. [32] generated an effective cellular immune response with the plasmid encoding the envelope glycoprotein gp51 and the transmembrane glycoprotein gp30 of the bovine leukemia virus (BLV), and a DNA vaccine encoding the fusion (F) gene of bovine respiratory syncytial virus (BRSV) induced protection against the infection in calves. Protective DNA vaccine strategies were identified against bovine herpes virus-1 (BHV-1) infection [33,34], and DNA immunization using the gC gene of BHV-1 induced both neutralizing antibodies and lympho-proliferative responses in bovines [35].

Small Ruminants: DNA vaccine candidates were developed for preventing viral diseases of small ruminants. Thus, protection was obtained against diseases like caprine arthritis-encephalitis (CAE) [77], foot and mouth disease (FMD) [78], and Visna-Maedi [79] and Rift valley fever [80]. Sheep vaccination with a DNA vaccine encoding the hepatitis B virus surface antigen (HBsAg) was as efficient as the commercial protein subunit vaccine for induction of long-term protective antibody titers against hepatitis B virus [25]. 

Swine: In swine, plasmid DNA vaccines could protect against classical swine fever (CSF), which causes significant losses to the pig industry [51,52]. Immunization with a plasmid encoding the E2 protein of CSF virus conferred protection against viral challenge, and the co-delivery of IL-3, IL-18, and CD 154 as adjuvants further enhanced the protection [51,52,53]. The viral protein genes such as those coding for gB, gC, and gD have been used for constructing DNA vaccine candidates against pseudorabies (Aujesky’s disease) in swine [54].

Dogs: For dogs the important viral diseases are rabies, canine distemper, and parvoviral infections, and many groups have studied the use of DNA vaccines. For example, DNA vaccines encoding the rabies virus glycoprotein (G) induced strong and durable virus-neutralizing antibody titers in dogs [36,37]. A bicistronic multivalent DNA vaccine comprised of rabies virus (G) and parvovirus (VP2) genes could induce neutralizing antibodies against both viral pathogens [28].

Horses: In horses DNA vaccine constructs encoding the envelope proteins (prM and E) have prevented the West Nile virus (WNV) infection [38], and as discussed below in more detail, an equine WNV vaccine was licensed in the US. Effective DNA vaccine candidates encoding the ORFs (5 and 7) of Equine arteritis virus (EAV) were also developed [40]. 

Poultry: In poultry, DNA vaccine candidates have been studied against major viral infections like avian influenza, using the haemagglutinin (HA) gene [41,42]. Similarly, a DNA vaccine encoding the fusion (F) and (HA) genes induced high levels of antibodies against Newcastle disease (NDV) in chickens [43]. Plasmid DNA vaccines encoding the N protein and the S1 glycoprotein genes of infectious bronchitis virus have been pursued [44]. Against infectious bursal disease (IBD), plasmid DNA encoding the VP2 gene and VP2/4/3 poly-protein genes of IBD virus have been used in protective DNA vaccine trials [45]. Against chicken infectious anemia (CIA), the use of the viral proteins VP1 and VP2 as proteins and as DNA vaccines have induced protective antibodies [46]. 

## 3. Licensed Veterinary Plasmid DNA Vaccines

In various animal species the plasmid DNA vaccines have worked very well, and plasmid DNA vaccines have been approved and licensed for veterinary use. Three plasmid DNA vaccines for prevention of infectious diseases have received approval, including two for fish and one for horses. These included two for fish (salmon) [19]: one for infectious hematopoietic necrosis virus (in 2005) [20], and one for pancreas disease (in 2016) caused by salmon alphavirus subtype 3 [21]. An equine vaccine co-developed by the US CDC and Fort Dodge Animal Health against the West Nile virus (WNV) was licensed (in 2005) [39]. This vaccine was also used in various avian species (robins, crows, and Western scrub-jays) [47,48,49,50] because of the central role that various wild birds play in the geographic spread and transmission cycle of WNV for mosquitos which then infect humans. Significantly, the vaccine was administered to California condors, with the US CDC subsequently attributing the prevention of extinction of this endangered species as the WNV spread westward across the USA [81]. A fourth DNA vaccine to treat cancer was initially approved for melanoma for dogs in the US (in 2010). However, because historic controls had been used in the efficacy study, when the European Medicines Agency request additional information, the company decided to change priorities rather than invest in the additional required studies [82]. Another plasmid DNA product has been licensed for therapeutic purposes for pigs raised for human consumption. The plasmid encodes growth hormone releasing hormone (GHRH), and is used for pregnant sows resulting in larger litters, both in terms of numbers of piglets and their birthweights. The plasmid is delivered using an electroporation device [83,84]. 

## 4. Rationale for Further Development and Use of DNA Vaccines for Animal Use

Given the strong effect in animals, the marked advantages of NA vaccines, and the increase in new virus threats for animals and in cases of zoonotic viruses for humans, the development and usage of more preventive NA vaccines in animals, especially in large flocks of production animals, is important.

The purpose of vaccinations is twofold: to protect the individual and to protect the flock. The individual argument in humans is influenced by some individual-specific risk factors relevant for the different virus diseases, e.g., chronic lung disease as a risk factor for Covid-19 infection, which affect the risk/benefit ratio for any disease and its prophylactic vaccine. Protection of a flock or a population may be determined by certain health measures and political strategies. For production animals, protection of the herd seems more relevant than the individual animal. In addition, protection by vaccines against infectious diseases may lower the general use of antibiotics and improve production in general, e.g., decrease the need to slaughter chickens during influenza outbreaks [85]. These factors may influence the choice of vaccine and vaccine strategy. In general, NA vaccines induced both antibodies to protect from infection and CMI to protect from disease and death. The design and components included in a particular NA vaccine mix may differ depending of the purpose. In addition, two different vaccines may be preferred for animals and humans for the same zoonotic virus. These may differ in design, production, quality, delivery method, cost, availability, and recommendation for use. 

For these reasons, two different designs of a broad protective influenza NA vaccine candidate may be considered. The version of a broad influenza DNA vaccine for production pigs may induce mostly antibodies (against HA and/or NA) to prevent infection and spread by shedding of influenza virus. In contrast, the version for humans should also contain genes for conserved internal virus proteins for additional CMI induction (against M and/or NP) to protect humans from disease and death, as well [66,86,87], and to provide potential cross-strain protection against newly arising drifted and shifted strains [5].

For DNA vaccines, there is a dose–response correlation, and in some cases a higher DNA dose is needed for obtaining cross-protection against variant viruses [86]. In addition, there is a species-specific sensitivity for DNA vaccines, e.g., the amount of plasmid DNA required in general for IM administration is 10–100 μg in mice, 100–300 μg in small animals, and 0.5–2.5 mg in larger animals like non-human primates and humans.

Efficacy of DNA Vaccines in Target Animals vs. Animal Models: The development and testing for efficacy and relevant dose of NA vaccines is much easier using exclusively the actual target animal. Although the best animal model for human influenza and thus influenza vaccine candidates are ferrets it may not be the best model for a DNA vaccine since DNA works very well in ferrets but perhaps suboptimally in primates like humans. In contrast, the best model for influenza NA vaccines for pigs may be pigs. Plasmid DNA vaccines work extremely well in pigs and only one vaccination of a very low DNA dose can protect pigs from influenza challenge [86,87,88]. Moreover, DNA vaccines have been shown to induce active immunity in piglets in the presence of maternal antibodies that otherwise prevent the use of protein vaccine until the absence of maternal antibodies. In addition, it may be easier to obtain a “lifelong” vaccine protection in production pigs since the lifetime of a production pig is less than one year. 

Delivery Modalities: The delivery of a plasmid DNA vaccine and vaccine organ may be crucial for its immunogenicity, optimal dose, schedule, and effect. Whereas intradermal injection followed by electroporation was earlier shown to be superior to just needle injection, especially in case of a suboptimal working DNA vaccine [89,90,91,92], it has been found that jet-injection to skin, which is a very immunogenic organ, results in the same optimal immune induction in animals like rabbits, ferrets and pigs [93]. This is crucial since jet-injection has several practical advantages in case of mass-vaccination of flocks of 10,000 pigs. Jet-injection is fast (a fraction of a second), less painful than electroporation, and can save ca. 5 min of constraining of the pig, which saves expensive time, improves animal welfare, and prevents needle accidents [86]. 

In addition, alternative delivery methods like suppositories [33], mucosal delivery [94,95], and topical application [23], have been found useful. Vaccination using nanoparticle-mediated DNA plasmid or mRNA delivery has been used for both protecting the nucleic acids and augmenting the immune responses due to better uptake and in some cases, potential adjuvant effects. In addition, co-delivery of plasmid-encoding cytokines (e.g., IFN-alpha, IL-15, IL-12) are used as NA vaccine adjuvants [96,97,98].

The vaccine cost, including the actual time per animal needed to perform the immunization, is among the most crucial parameters for determining the use of a safe and efficient NA vaccine in production animals. Depending on the disease and country, a vaccine for production animals should probably cost less than one dollar. Although plasmid production in *E. coli* is fast and simple, this likely requires an in-house GMP DNA vaccine production facility of large capacity with QA and filling, since at present, most Clinical Manufacturing Organization prices exceed this. However, effective, safe, and economically viable nucleic acid vaccines for animal use are indeed possible and needed.

## 5. Human Clinical Trials

A number of the early targets for DNA vaccines are focused on diseases for which traditional approaches have not yielded successful vaccines, such as HIV and malaria, but also cancer in the form of immunotherapeutic vaccines as treatments. The ability to generate Cytotoxic T Lymphocyte responses (CTL) by DNA vaccines was a significant reason for the excitement because CTL are thought to be important for controlling diseases by pathogens that invade cells (e.g., viruses, mycobacteria, parasites) and cancer. CTL responses cannot prevent initial infection, since CTLs can only kill virally infected cells, but not directly viruses, but this activity is important in many viral infections for limiting disease even if CTLs cannot provide “sterilizing immunity” against initial infection. (CTLs are important for killing tumor cells both for immune surveillance for controlling the growth of tumors that arise spontaneously, but also as evidenced by the immense success of immune-oncologic approaches such as Car-T cells.) Importantly for viral infections, CTLs can target epitopes from proteins that are more highly conserved such as viral enzymes or other internal proteins, which are less likely to mutate (since they must conserve their function or structure) than are surface proteins. Excellent examples of this are the diversity and mutations seen for influenza hemagglutinin and HIV Env, compared to, for example, influenza Nucleoprotein or HIV Gag. This makes the role of CTL potentially of great importance for an immune response that can be effective against different strains of a virus, such as those occurring for influenza with the annual shifts and periodic drifts of the strains (i.e., smaller or larger mutations that obviate the protection of strain-specific antibodies).

Results of Select Illustrative Human Clinical Trials of DNA Vaccines: Relevance of Veterinary Successes: The human clinical trials of DNA vaccines for many different targets, and by having been in thousands of people [5,11], have increased the confidence in their safety. Unfortunately, they also demonstrated that with a few exceptions, the DNA vaccines, while generating antibodies, T cell help, and CTLS, were not generally potent enough to become approved human vaccines. It is not clear whether this insufficient potency was due to the targets (such as HIV, where any correlates of efficacy are not known) or to the technology itself or some combination. It was long thought that the issue was simply that the mass of animals for the pre-clinical trials (generally mice, ferrets) was so small compared to humans, that the amount of antigen produced was insufficient for potency when moving into humans. Moreover, since many vaccines work via antibody responses, at least in terms of demonstrating a correlate of efficacy, DNA vaccines, while capable of stimulating antibodies, are considered more effective for CTLs and T helper cells than for antibody responses. 

Thus, the licensure of a plasmid DNA vaccine for West Nile virus for horses [39] and the responses seen in cattle for bovine herpes virus [99,100,101] were significant for showing that dosing/mass was not an absolute barrier. The WNV vaccine results also pointed towards the importance of the antigen being a highly immunogenic protein, since one difference between the WNV vaccine and other vaccines is that the WNV plasmid vaccine encodes both the premembrane (prM) and surface envelope (E) antigens which form a subviral particle that is virus-like when produced in cells, with the resulting structure having antigenic properties like the regular infectious virion [12]. The WNV plasmid DNA for the human trials was analogous to the one used in horses, and included design elements to maximize the in vivo protein production. The human clinical results demonstrated that after three doses, the neutralizing antibody titers were comparable to protective titers in horses [12] even in older humans who generally have poorer immune responses to vaccines [15]. Based on these encouraging results, when Ebola and Zika viruses emerged, plasmid DNA vaccines were quickly constructed, and after promising pre-clinical data, were clinically tested in humans. The Ebola vaccine was found to be immunogenic with a three-dose regimen [102], as were Zika vaccines delivered either with electroporation [103] or without [14]. However, in the case of Ebola, a viral vectored vaccine that was effective with a single shot was ultimately licensed [104]. 

While plasmid DNA vaccines generated immune responses in a variety of vaccine trials, the challenge for demonstrating human efficacy appeared to be in part due to the limitation of needing up to three doses to generate sufficient neutralizing antibody. In the case of vaccines where CTLs were thought to be important such as for HIV, malaria, and Tb, at least part of the challenge is that the necessary protective immune responses, and any correlates for cellular responses, are not really known and the diseases themselves have additional challenges compared to, e.g., WNV, in that HIV integrates, and for Tb, as the mycobacterium can infect cells latently. Thus, the problem appears to be a combination of lack of potency for the DNA vaccines combined with the difficulty of knowing exactly what immune responses are needed and the difficulty of diseases for which most of the clinical trials have been done.

Potency of DNA vaccines as Priming for Heterologous Prime-Boost Regimens: One of the most interesting observations has been that plasmid DNA vaccines work extremely well as a priming agent for heterologous prime-boosting protocols in which plasmid DNA encoding an antigen is given initially, with a subsequent boost being a different form of the antigen, whether a recombinant protein, or even a viral vector encoding the same antigen (reviewed in [105]). This results in extremely high titers of antibodies. One mechanism for this is thought to be that in comparison to a viral vector homologous prime/boost, where much of the initial immune response is directed against the vector, and where the vector given as a boost may then be targeted by immune responses directed against the vector (thus decreasing the production of vector-encoded antigen, and hence decreasing the effectiveness of the boost), with a DNA prime, the priming response is only against the encoded antigen. Then, when a recombinant protein or a viral vector boost is given, which can result in a larger amount of antigen being made, only the B cell responses directed against the antigen are boosted. The high titers of antibody response are seen only when the prime is DNA, not the other order, and the immune responses generally are even greater with a heterologous prime boost than if, for example, a viral vector is given twice. This point is being raised because of what is being clinically proposed for the roll-out of vaccines in the UK, and possibly other countries, with the different types of Covid-19 vaccines: despite the phase-3 efficacy trials being done for each vaccine independently (i.e., the booster doses are of the same vaccine that was initially administered to a person), it appears that clinicians may be allowed to use different vaccines for the prime and the boost. The vaccines either approved for emergency use authorization or those expected to be soon approved are either mRNA nucleic acid vaccines or viral vectors (adenovirus vector and chimp adenovirus vector). It is of course unknown if an mRNA vaccine prime followed by a viral vector boost will provide the same high immune responses as with plasmid DNA prime followed by viral vector boost, but based on the putative mechanism, and given the high degree of innate stimulation also contributed by the mRNA vaccine and possibly their lipid nanoparticle formulations, such a prime boost might be very potent, but the reverse order might not if the results are similar to what has been seen with plasmid DNA vaccines. Certainly, such off-label use of the vaccines should be carefully studied before endorsing this approach, particularly if giving the NA vaccine as the boost to another modality results in a lower immune response.

One obvious advantage of NA vaccines, whether plasmid DNA or mRNA, is the speed with which they can be designed and redesigned (if needed in case of mutations if those mutations change the epitopes for neutralizing antibody), constructed and manufactured. This is because either can be easily made from knowledge of the gene sequence of the pathogen without having to deal with the actual pathogen itself. Of course, understanding aspects of the antigenicity is important, such as the approach for SARS-CoV-2 vaccines where the pre-fusion form of the spike protein is thought to be key [106]. The manufacture of both plasmid DNA and mRNA is essentially generic, and is much simpler than having to attenuate a virus and then determine how to grow the virus, or compared to a viral vector or even a recombinant protein. While plasmid DNA is quite stable, thus being appealing for globally-needed vaccines, to date, the mRNA vaccines made are more fragile than plasmid DNA, so for global usage, one current challenge of the two authorized mRNA vaccines is the transport and storage cold chain requirements, with one vaccine requiring −70 °C freezers that are not typical equipment for healthcare providers. However, progress is being made for thermostable mRNA vaccines [107].

## 6. Nucleic Acid Vaccines in the Context of One Health

Two key aspects stand out for nucleic acid vaccines in the context of One Health: vaccines for infectious diseases whose spread involves both animal and human hosts (such as the West Nile virus discussed above) and vaccines for diseases which may arise as new species in animals which then cross into humans, as apparently happened with SARS-CoV-2. For the former, scientific progress will advance much more rapidly with more collaborations between vaccinologists who focus on animal and human vaccines. There are many areas of overlap in addition to the virus-specific aspects of immunity. For example, given the size of pigs and cattle, the lessons learned for potency will possibly be more synergistic than trying to predict from murine studies how well a vaccine will work in humans. The usage of biolistics devices with delivery to pig skin is another example where there may be relevance for human i.d. delivery and vice versa. Additionally, of course, a number of pathogens are specifically shared or are very similar between humans and animals, whether viruses such as influenza, parasites, or mycobacteria. 

The other urgent One Health aspect that drives the development of rapid-deployment technologies such as NA vaccines are the emerging diseases that cross from animal species to humans (and then back in the other direction as has been seen with SARS-CoV-2 in mink farms). Of course, development of platform technologies such as nucleic acid vaccines is only half of the equation, as the ability to respond quickly to any new pathogen will also require expanded surveillance efforts and the infrastructure for massive vaccine delivery campaigns both for humans and animals. 

It is clear that Nucleic Acid vaccines are a platform technology for new emergency vaccines, both in terms of design and manufacture. While for humans this has only been proven through Phase 3 efficacy trials for mRNA vaccines, the speed and early clinical trial results for DNA vaccines (but that generally required three doses to achieve desired titers [12,14,15,102,103]) and the licensed veterinary DNA vaccines (see above) demonstrate that nucleic acid vaccines are a powerful approach. One cannot help but wonder whether if the focus for plasmid DNA had been more on optimizing delivery rather than on diseases as challenging as HIV, malaria, and Tb, the technology would already be used more for human vaccines other than the handful that are licensed for veterinary use. Nevertheless, what has been learned through all the preclinical and clinical studies of plasmid DNA, in terms of immune responses that can develop when an antigen is made in situ, and gaining the clinical experience in terms of safety (i.e., no autoimmunity or tolerance), has contributed immensely to the rapid demonstration of efficacy of mRNA vaccines against Covid-19. With the successful mRNA vaccines and the Sars-CoV-2/Covid19 plasmid DNA vaccines in the pipeline, it is expected that considerable experience and acceptance of NA vaccines will emerge. 

What must continue to be studied as these technologies are used more and more, are the One Health aspects of disease emergence, containment, and immunization, remembering that humans are but one part of the ecosystem. Large data sets are being generated now for NA vaccines’ safety, efficacy and effectiveness, optimal doses, vaccination scheme, prime-boost use, and possible correlates of protection against sars-Cov-2/Covid-19 infection and disease. In addition, the WHO has developed, with the input of regulators worldwide, guidelines for DNA vaccines [108]. All these will facilitate the vaccine revolution NA vaccines have the potential to be.

## Figures and Tables

**Table 1:** Selected nucleic acid vaccines evaluated, in development, or licensed, for viral veterinary medicine.

Animal	Pathogen	Vaccine Gene	Response	References
Bovine	Rotavirus	VP4	Th1 immune response	[31]
	Leukemia virus	gp30, gp51	Cell-mediated immunity	[32]
	Respiratory syncytial virus	Fusion (F) protein	Protection from infection	[29]
	Herpes virus-1	gD; gC	Protection from infection, neutralizing antibodies, lymphoproliferative response	[33,34,35]
Sheep	Hepatitis B virus	HBsAg	Protective antibodies	[25]
Dog	Rabies virus	GP	Neutralizing antibodies	[28,36,37]
	Parvovirus	VP2		[28]
Horse	West Nile virus *	prM, E	Protection from infection	[38,39]
	Equine arteritis virus	Orf 5 and 7	Protection from infection	[40]
Birds	Avian influenza virus	HA	Protection	[41,42]
	Newcastle disease	HA and F	Antibodies	[43]
	Infectious bronchitis virus	N and S1 GP	CTL, protection from disease	[44]
	Infectious bursal disease	VP2, VP2/4/3	Protection from infection	[45]
	Chicken infectious anemia	VP1, VP2	Protective antibodies	[46]
	West Nile virus	prM, E	Protection from infection	[47,48,49,50]
Swine	Classical swine fever	E2	Protection from infection	[51,52,53]
	Pseudorabies (Aujesky’s disease)	Pv gB, gC, gD	Protection from infection	[54]
Fish	Hematopoietic necrosis virus *	Glycoprotein	Protection from infection	[20]
	Salmon alphavirus subtype 3 *	Salmon alphavirus proteins	Protection from infection	[21]

* Licensed vaccine.

## Data Availability

Not applicable.

## References

[1] Wolff J.A., Malone R.W., Williams P., Chong W., Acsadi G., Jani A., Felgner P.L. (1990). Direct Gene Transfer into Mouse Muscle in Vivo. Science.

[2] Ulmer J.B., Donnelly J.J., Parker S.E., Rhodes G.H., Felgner P.L., Dwarki V.J., Gromkowski S.H., Deck R.R., DeWitt C.M., Friedman A. (1993). Heterologous Protection against Influenza by Injection of DNA Encoding a Viral Protein. Science.

[3] Donnelly J.J., Wahren B., Liu M.A. (2005). DNA Vaccines: Progress and Challenges. J. Immunol..

[4] Fu T.-M., Ulmer J.B., Caulfield M.J., Deck R.R., Friedman A., Wang S., Liu X., Donnelly J.J., Liu M.A., Wigzell H. (1997). Priming of Cytotoxic T Lymphocytes by DNA Vaccines: Requirement for Professional Antigen Presenting Cells and Evidence for Antigen Transfer from Myocytes. Mol. Med..

[5] Liu M.A. (2011). DNA Vaccines: An Historical Perspective and View to the Future. Immunol. Rev..

[6] Tang C.-K., Pietersz G.A. (2009). Intracellular Detection and Immune Signaling Pathways of DNA Vaccines. Expert Rev. Vaccines.

[7] Liu M.A. (2019). A Comparison of Plasmid DNA and MRNA as Vaccine Technologies. Vaccines.

[8] Ledwith B.J., Manam S., Troilo P.J., Barnum A.B., Pauley C.J., Ii T.G.G., Harper L.B., Beare C.M., Bagdon W.J., Nichols W.W. (2000). Plasmid DNA Vaccines: Investigation of Integration into Host Cellular DNA Following Intramuscular Injection in Mice. Intervirology.

[9] Gottlieb P., Utz P.J., Robinson W., Steinman L. (2013). Clinical Optimization of Antigen Specific Modulation of Type 1 Diabetes with the Plasmid DNA Platform. Clin. Immunol..

[10] Garren H., Robinson W.H., Krasulová E., Havrdová E., Nadj C., Selmaj K., Losy J., Nadj I., Radue E.-W., Kidd B.A. (2008). Phase 2 Trial of a DNA Vaccine Encoding Myelin Basic Protein for Multiple Sclerosis. Ann. Neurol..

[11] Liu M.A., Ulmer J.B. (2005). Human Clinical Trials of Plasmid DNA Vaccines. Adv. Genet..

[12] Martin J.E., Pierson T.C., Hubka S., Rucker S., Gordon I.J., Enama M.E., Andrews C.A., Xu Q., Davis B.S., Nason M.C. (2007). A West Nile Virus DNA Vaccine Induces Neutralizing Antibody in Healthy Adults during a Phase 1 Clinical Trial. J. Infect. Dis..

[13] Sarwar U.N., Costner P., Enama M.E., Berkowitz N., Hu Z., Hendel C.S., Sitar S., Plummer S., Mulangu S., Bailer R.T. (2015). Safety and Immunogenicity of DNA Vaccines Encoding Ebolavirus and Marburgvirus Wild-Type Glycoproteins in a Phase I Clinical Trial. J. Infect. Dis..

[14] Gaudinski M.R., Houser K.V., Morabito K.M., Hu Z., Yamshchikov G., Rothwell R.S., Berkowitz N., Mendoza F., Saunders J.G., Novik L. (2018). Safety, Tolerability, and Immunogenicity of Two Zika Virus DNA Vaccine Candidates in Healthy Adults: Randomised, Open-Label, Phase 1 Clinical Trials. Lancet.

[15] Ledgerwood J.E., Pierson T.C., Hubka S.A., Desai N., Rucker S., Gordon I.J., Enama M.E., Nelson S., Nason M., Gu W. (2011). A West Nile Virus DNA Vaccine Utilizing a Modified Promoter Induces Neutralizing Antibody in Younger and Older Healthy Adults in a Phase I Clinical Trial. J. Infect. Dis..

[16] Kim T.J., Jin H.-T., Hur S.-Y., Yang H.G., Seo Y.B., Hong S.R., Lee C.-W., Kim S., Woo J.-W., Park K.S. (2014). Clearance of Persistent HPV Infection and Cervical Lesion by Therapeutic DNA Vaccine in CIN3 Patients. Nat. Commun..

[17] Chudley L., McCann K., Mander A., Tjelle T., Campos-Perez J., Godeseth R., Creak A., Dobbyn J., Johnson B., Bass P. (2012). DNA Fusion-Gene Vaccination in Patients with Prostate Cancer Induces High-Frequency CD8(+) T-Cell Responses and Increases PSA Doubling Time. Cancer Immunol. Immunother. CII.

[18] Patel P.M., Ottensmeier C.H., Mulatero C., Lorigan P., Plummer R., Pandha H., Elsheikh S., Hadjimichael E., Villasanti N., Adams S.E. (2018). Targeting Gp100 and TRP-2 with a DNA Vaccine: Incorporating T Cell Epitopes with a Human IgG1 Antibody Induces Potent T Cell Responses That Are Associated with Favourable Clinical Outcome in a Phase I/II Trial. Oncoimmunology.

[19] Dalmo R.A. (2018). DNA Vaccines for Fish: Review and Perspectives on Correlates of Protection. J. Fish Dis..

[20] Garver K.A., LaPatra S.E., Kurath G. (2005). Efficacy of an Infectious Hematopoietic Necrosis (IHN) Virus DNA Vaccine in Chinook Oncorhynchus Tshawytscha and Sockeye O. Nerka Salmon. Dis. Aquat. Organ..

[21] Thorarinsson R., Wolf J.C., Inami M., Phillips L., Jones G., Macdonald A.M., Rodriguez J.F., Sindre H., Skjerve E., Rimstad E. (2021). Effect of a Novel DNA Vaccine against Pancreas Disease Caused by Salmonid Alphavirus Subtype 3 in Atlantic Salmon (Salmo Salar). Fish Shellfish Immunol..

[22] Dunham S.P. (2002). The Application of Nucleic Acid Vaccines in Veterinary Medicine. Res. Vet. Sci..

[23] Oshop G.L., Elankumaran S., Heckert R.A. (2002). DNA Vaccination in the Avian. Vet. Immunol. Immunopathol..

[24] Babiuk L.A., Pontarollo R., Babiuk S., Loehr B., van Drunen Littel-van den Hurk S. (2003). Induction of Immune Responses by DNA Vaccines in Large Animals. Vaccine.

[25] Babiuk S., Tsang C., van Drunen Littel-van den Hurk S., Babiuk L.A., Griebel P.J. (2007). A Single HBsAg DNA Vaccination in Combination with Electroporation Elicits Long-Term Antibody Responses in Sheep. Bioelectrochem. Amst. Neth..

[26] Ding X., Lillehoj H.S., Dalloul R.A., Min W., Sato T., Yasuda A., Lillehoj E.P. (2005). In Ovo Vaccination with the Eimeria Tenella EtMIC2 Gene Induces Protective Immunity against Coccidiosis. Vaccine.

[27] Gupta P.K., Sharma S., Walunj S.S., Patil A.A., Rai A., Saini M. (2006). A DNA Vaccine That Encodes Rabies Virus Glycoprotein Lacking Transmembrane Domain Enhances Antibody Response but Not Protection. Acta Virol..

[28] Patial S., Chaturvedi V.K., Rai A., Saini M., Chandra R., Saini Y., Gupta P.K. (2007). Virus Neutralizing Antibody Response in Mice and Dogs with a Bicistronic DNA Vaccine Encoding Rabies Virus Glycoprotein and Canine Parvovirus VP2. Vaccine.

[29] Dhama K., Mahendran M., Gupta P.K., Rai A. (2008). DNA Vaccines and Their Applications in Veterinary Practice: Current Perspectives. Vet. Res. Commun..

[30] Min W., Lillehoj H.S., Burnside J., Weining K.C., Staeheli P., Zhu J.J. (2001). Adjuvant Effects of IL-1beta, IL-2, IL-8, IL-15, IFN-Alpha, IFN-Gamma TGF-Beta4 and Lymphotactin on DNA Vaccination against Eimeria Acervulina. Vaccine.

[31] Suradhat S., Yoo D., Babiuk L.A., Griebel P., Baca-Estrada M.E. (1997). DNA Immunization with a Bovine Rotavirus VP4 Gene Induces a Th1-like Immune Response in Mice. Viral Immunol..

[32] Brillowska A., Dabrowski S., Rułka J., Kubiś P., Buzała E., Kur J. (1999). Protection of Cattle against Bovine Leukemia Virus (BLV) Infection Could Be Attained by DNA Vaccination. Acta Biochim. Pol..

[33] Loehr B.I., Rankin R., Pontarollo R., King T., Willson P., Babiuk L.A., van Drunen Littel-van den Hurk S. (2001). Suppository-Mediated DNA Immunization Induces Mucosal Immunity against Bovine Herpesvirus-1 in Cattle. Virology.

[34] Castrucci G., Ferrari M., Salvatori D., Sardonini S., Frigeri F., Petrini S., Lo Dico M., Marchini C., Rotola A., Amici A. (2005). Vaccination Trials against Bovine Herpesvirus-1. Vet. Res. Commun..

[35] Gupta P.K., Saini M., Gupta L.K., Rao V.D., Bandyopadhyay S.K., Butchaiah G., Garg G.K., Garg S.K. (2001). Induction of Immune Responses in Cattle with a DNA Vaccine Encoding Glycoprotein C of Bovine Herpesvirus-1. Vet. Microbiol..

[36] Rai N., Kaushik P., Rai A. (2005). Development of Rabies DNA Vaccine Using a Recombinant Plasmid. Acta Virol..

[37] Gupta P.K., Rai A., Rai N., Saini M. (2005). Immunogenicity of a Recombinant Plasmid DNA Containing Glycoprotein Gene of Rabies Virus CVS. J. Immunol. Immunopathol..

[38] Hall R.A., Khromykh A.A. (2004). West Nile Virus Vaccines. Expert Opin. Biol. Ther..

[39] CDC—Media Relations—Press Release—18 July 2005. https://www.cdc.gov/media/pressrel/r050718.htm.

[40] Giese M., Bahr U., Jakob N.J., Kehm R., Handermann M., Müller H., Vahlenkamp T.H., Spiess C., Schneider T.H., Schusse G. (2002). Stable and Long-Lasting Immune Response in Horses after DNA Vaccination against Equine Arteritis Virus. Virus Genes.

[41] Kodihalli S., Kobasa D.L., Webster R.G. (2000). Strategies for Inducing Protection against Avian Influenza A Virus Subtypes with DNA Vaccines. Vaccine.

[42] Lee C.-W., Senne D.A., Suarez D.L. (2004). Generation of Reassortant Influenza Vaccines by Reverse Genetics That Allows Utilization of a DIVA (Differentiating Infected from Vaccinated Animals) Strategy for the Control of Avian Influenza. Vaccine.

[43] Loke C.F., Omar A.R., Raha A.R., Yusoff K. (2005). Improved Protection from Velogenic Newcastle Disease Virus Challenge Following Multiple Immunizations with Plasmid DNA Encoding for F and HN Genes. Vet. Immunol. Immunopathol..

[44] Seo S.H., Wang L., Smith R., Collisson E.W. (1997). The Carboxyl-Terminal 120-Residue Polypeptide of Infectious Bronchitis Virus Nucleocapsid Induces Cytotoxic T Lymphocytes and Protects Chickens from Acute Infection. J. Virol..

[45] Li L., Fang W., Li J., Huang Y., Yu L. (2006). Oral DNA Vaccination with the Polyprotein Gene of Infectious Bursal Disease Virus (IBDV) Delivered by the Attenuated Salmonella Elicits Protective Immune Responses in Chickens. Vaccine.

[46] Senthil Kumar N., Kataria J.M., Dhama K., Bhardwaj N., Sylvester S.A., Rahul S. Development of DNA Vaccine against Chicken Anemia Virus Simultaneously Using It’s VP1 and VP2 Proteins. Proceedings of the XII Conference of IPSA and National Symposium Himachal Pradesh Krishi Vishwavidalya.

[47] Kilpatrick A.M., Dupuis A.P., Chang G.-J.J., Kramer L.D. (2010). DNA Vaccination of American Robins (Turdus Migratorius) against West Nile Virus. Vector Borne Zoonotic Dis. Larchmt. N.

[48] Bunning M.L., Fox P.E., Bowen R.A., Komar N., Chang G.-J.J., Speaker T.J., Stephens M.R., Nemeth N., Panella N.A., Langevin S.A. (2007). DNA Vaccination of the American Crow (Corvus Brachyrhynchos) Provides Partial Protection against Lethal Challenge with West Nile Virus. Avian Dis..

[49] Turell M.J., Bunning M., Ludwig G.V., Ortman B., Chang J., Speaker T., Spielman A., McLean R., Komar N., Gates R. (2003). DNA Vaccine for West Nile Virus Infection in Fish Crows (Corvus Ossifragus). Emerg. Infect. Dis..

[50] Wheeler S.S., Langevin S., Woods L., Carroll B.D., Vickers W., Morrison S.A., Chang G.-J.J., Reisen W.K., Boyce W.M. (2011). Efficacy of Three Vaccines in Protecting Western Scrub-Jays (Aphelocoma Californica) from Experimental Infection with West Nile Virus: Implications for Vaccination of Island Scrub-Jays (Aphelocoma Insularis). Vector Borne Zoonotic Dis. Larchmt. N.

[51] Wienhold D., Armengol E., Marquardt A., Marquardt C., Voigt H., Büttner M., Saalmüller A., Pfaff E. (2005). Immunomodulatory Effect of Plasmids Co-Expressing Cytokines in Classical Swine Fever Virus Subunit Gp55/E2-DNA Vaccination. Vet. Res..

[52] Andrew M., Morris K., Coupar B., Sproat K., Oke P., Bruce M., Broadway M., Morrissy C., Strom D. (2006). Porcine Interleukin-3 Enhances DNA Vaccination against Classical Swine Fever. Vaccine.

[53] Li N., Qiu H.-J., Zhao J.-J., Li Y., Wang M.-J., Lu B.-W., Han C.-G., Hou Q., Wang Z.-H., Gao H. (2007). A Semliki Forest Virus Replicon Vectored DNA Vaccine Expressing the E2 Glycoprotein of Classical Swine Fever Virus Protects Pigs from Lethal Challenge. Vaccine.

[54] Dory D., Béven V., Torché A.-M., Bougeard S., Cariolet R., Jestin A. (2005). CpG Motif in ATCGAT Hexamer Improves DNA-Vaccine Efficiency against Lethal Pseudorabies Virus Infection in Pigs. Vaccine.

[55] Hui D.S.C., Chan P.K.S. (2010). Severe Acute Respiratory Syndrome and Coronavirus. Infect. Dis. Clin. North Am..

[56] Neumann G., Noda T., Kawaoka Y. (2009). Emergence and Pandemic Potential of Swine-Origin H1N1 Influenza Virus. Nature.

[57] Gire S.K., Goba A., Andersen K.G., Sealfon R.S.G., Park D.J., Kanneh L., Jalloh S., Momoh M., Fullah M., Dudas G. (2014). Genomic Surveillance Elucidates Ebola Virus Origin and Transmission during the 2014 Outbreak. Science.

[58] Liu Y., Liu J., Du S., Shan C., Nie K., Zhang R., Li X.-F., Zhang R., Wang T., Qin C.-F. (2017). Evolutionary Enhancement of Zika Virus Infectivity in Aedes Aegypti Mosquitoes. Nature.

[59] Andersen K.G., Rambaut A., Lipkin W.I., Holmes E.C., Garry R.F. (2020). The Proximal Origin of SARS-CoV-2. Nat. Med..

[60] Zhou P., Yang X.-L., Wang X.-G., Hu B., Zhang L., Zhang W., Si H.-R., Zhu Y., Li B., Huang C.-L. (2020). A Pneumonia Outbreak Associated with a New Coronavirus of Probable Bat Origin. Nature.

[61] Hammer A.S., Quaade M.L., Rasmussen T.B., Fonager J., Rasmussen M., Mundbjerg K., Lohse L., Strandbygaard B., Jørgensen C.S., Alfaro-Núñez A. (2021). Early Release—SARS-CoV-2 Transmission between Mink (Neovison Vison) and Humans, Denmark. Emerg. Infect. Dis. J. CDC.

[62] Nelson M.I., Gramer M.R., Vincent A.L., Holmes E.C. (2012). Global Transmission of Influenza Viruses from Humans to Swine. J. Gen. Virol..

[63] Sandbulte M.R., Spickler A.R., Zaabel P.K., Roth J.A. (2015). Optimal Use of Vaccines for Control of Influenza a Virus in Swine. Vaccines.

[64] WHO Avian and Other Zoonotic Influenza. http://www.who.int/influenza/human_animal_interface/en/.

[65] Bragstad K., Martel C.J., Thomsen J.S., Jensen K.L., Nielsen L.P., Aasted B., Fomsgaard A. (2011). Pandemic Influenza 1918 H1N1 and 1968 H3N2 DNA Vaccines Induce Cross-Reactive Immunity in Ferrets against Infection with Viruses Drifted for Decades. Influenza Other Respir. Viruses.

[66] Guilfoyle K., Major D., Skeldon S., James H., Tingstedt J.L., Polacek C., Lassauniére R., Engelhardt O.G., Fomsgaard A. (2020). Protective Efficacy of a Polyvalent Influenza A DNA Vaccine against Both Homologous (H1N1pdm09) and Heterologous (H5N1) Challenge in the Ferret Model. Vaccine.

[67] Borggren M., Nielsen J., Karlsson I., Dalgaard T.S., Trebbien R., Williams J.A., Fomsgaard A. (2016). A Polyvalent Influenza DNA Vaccine Applied by Needle-Free Intradermal Delivery Induces Cross-Reactive Humoral and Cellular Immune Responses in Pigs. Vaccine.

[68] Fischer L., Barzu S., Andreoni C., Buisson N., Brun A., Audonnet J.C. (2003). DNA Vaccination of Neonate Piglets in the Face of Maternal Immunity Induces Humoral Memory and Protection against a Virulent Pseudorabies Virus Challenge. Vaccine.

[69] Kitikoon P., Nilubol D., Erickson B.J., Janke B.H., Hoover T.C., Sornsen S.A., Thacker E.L. (2006). The Immune Response and Maternal Antibody Interference to a Heterologous H1N1 Swine Influenza Virus Infection Following Vaccination. Vet. Immunol. Immunopathol..

[70] Borggren M., Vinner L., Andresen B.S., Grevstad B., Repits J., Melchers M., Elvang T.L., Sanders R.W., Martinon F., Dereuddre-Bosquet N. (2013). Optimization of HIV-1 Envelope DNA Vaccine Candidates within Three Different Animal Models, Guinea Pigs, Rabbits and Cynomolgus Macaques. Vaccines.

[71] Shan S., Jiang Y., Bu Z., Ellis T., Zeng X., Edwards J., Tian G., Li Y., Ge J., Chen H. (2011). Strategies for Improving the Efficacy of a H6 Subtype Avian Influenza DNA Vaccine in Chickens. J. Virol. Methods.

[72] Williams J.A. (2013). Vector Design for Improved DNA Vaccine Efficacy, Safety and Production. Vaccines.

[73] Karikó K., Buckstein M., Ni H., Weissman D. (2005). Suppression of RNA Recognition by Toll-like Receptors: The Impact of Nucleoside Modification and the Evolutionary Origin of RNA. Immunity.

[74] Karikó K., Muramatsu H., Welsh F.A., Ludwig J., Kato H., Akira S., Weissman D. (2008). Incorporation of Pseudouridine into MRNA Yields Superior Nonimmunogenic Vector with Increased Translational Capacity and Biological Stability. Mol. Ther. J. Am. Soc. Gene Ther..

[75] Reichmuth A.M., Oberli M.A., Jaklenec A., Langer R., Blankschtein D. (2016). MRNA Vaccine Delivery Using Lipid Nanoparticles. Ther. Deliv..

[76] Karlsson I., Borggren M., Nielsen J., Christensen D., Williams J., Fomsgaard A. (2017). Increased Humoral Immunity by DNA Vaccination Using an α-Tocopherol-Based Adjuvant. Hum. Vaccines Immunother..

[77] Cheevers W.P., Snekvik K.R., Trujillo J.D., Kumpula-McWhirter N.M., Pretty On Top K.J., Knowles D.P. (2003). Prime-Boost Vaccination with Plasmid DNA Encoding Caprine-Arthritis Encephalitis Lentivirus Env and Viral SU Suppresses Challenge Virus and Development of Arthritis. Virology.

[78] Niborski V., Li Y., Brennan F., Lane M., Torché A.M., Remond M., Bonneau M., Riffault S., Stirling C., Hutchings G. (2006). Efficacy of Particle-Based DNA Delivery for Vaccination of Sheep against FMDV. Vaccine.

[79] Henriques A.M., Fevereiro M., Monteiro G.A. (2016). DNA Vaccines against Maedi-Visna Virus. Methods Mol. Biol..

[80] Gonzalez-Valdivieso J., Borrego B., Girotti A., Moreno S., Brun A., Bermejo-Martin J.F., Arias F.J. (2020). A DNA Vaccine Delivery Platform Based on Elastin-Like Recombinamer Nanosystems for Rift Valley Fever Virus. Mol. Pharm..

[81] Chang G.-J.J., Davis B.S., Stringfield C., Lutz C. (2007). Prospective Immunization of the Endangered California Condors (Gymnogyps Californianus) Protects This Species from Lethal West Nile Virus Infection. Vaccine.

[82] Anonymous Oncept Melanoma: Withdrawn Application. https://www.ema.europa.eu/en/medicines/veterinary/withdrawn-applications/oncept-melanoma.

[83] Draghia-Akli R., Ellis K.M., Hill L.-A., Malone P.B., Fiorotto M.L. (2003). High-Efficiency Growth Hormone-Releasing Hormone Plasmid Vector Administration into Skeletal Muscle Mediated by Electroporation in Pigs. FASEB J. Off. Publ. Fed. Am. Soc. Exp. Biol..

[84] VGX Animal Health Announces Approval of LifeTide^TM^ SW 5—World’s First and Only Approved DNA Therapy for Food Animals. https://vgxii.com/gx-animal-health-announces-approval-of-lifetide-sw-5-worlds-first-and-only-approved-dna-therapy-for-food-animalsgx-animal-health-announces-approval-of-lifetide-sw-5-worl/.

[85] Pasick J., Robinson J., Hooper-McGrevy K., Wright P., Kitching P., Handel K., Copps J., Ridd D., Kehler H., Hills K. (2007). The Roles of National and Provincial Diagnostic Laboratories in the Eradication of Highly Pathogenic H7N3 Avian Influenza Virus from the Fraser Valley of British Columbia, Canada. Avian Dis..

[86] Karlsson I., Borggren M., Rosenstierne M.W., Trebbien R., Williams J.A., Vidal E., Vergara-Alert J., Foz D.S., Darji A., Sisteré-Oró M. (2018). Protective Effect of a Polyvalent Influenza DNA Vaccine in Pigs. Vet. Immunol. Immunopathol..

[87] Sisteré-Oró M., López-Serrano S., Veljkovic V., Pina-Pedrero S., Vergara-Alert J., Córdoba L., Pérez-Maillo M., Pleguezuelos P., Vidal E., Segalés J. (2019). DNA Vaccine Based on Conserved HA-Peptides Induces Strong Immune Response and Rapidly Clears Influenza Virus Infection from Vaccinated Pigs. PLoS ONE.

[88] Gorres J.P., Lager K.M., Kong W.-P., Royals M., Todd J.-P., Vincent A.L., Wei C.-J., Loving C.L., Zanella E.L., Janke B. (2011). DNA Vaccination Elicits Protective Immune Responses against Pandemic and Classic Swine Influenza Viruses in Pigs. Clin. Vaccine Immunol..

[89] Otten G., Schaefer M., Doe B., Liu H., Srivastava I., zur Megede J., O’Hagan D., Donnelly J., Widera G., Rabussay D. (2004). Enhancement of DNA Vaccine Potency in Rhesus Macaques by Electroporation. Vaccine.

[90] Widera G., Austin M., Rabussay D., Goldbeck C., Barnett S.W., Chen M., Leung L., Otten G.R., Thudium K., Selby M.J. (2000). Increased DNA Vaccine Delivery and Immunogenicity by Electroporation in Vivo. J. Immunol..

[91] Hirao L.A., Wu L., Khan A.S., Satishchandran A., Draghia-Akli R., Weiner D.B. (2008). Intradermal/Subcutaneous Immunization by Electroporation Improves Plasmid Vaccine Delivery and Potency in Pigs and Rhesus Macaques. Vaccine.

[92] Bragstad K., Vinner L., Hansen M.S., Nielsen J., Fomsgaard A. (2013). A Polyvalent Influenza A DNA Vaccine Induces Heterologous Immunity and Protects Pigs against Pandemic A(H1N1)Pdm09 Virus Infection. Vaccine.

[93] Borggren M., Nielsen J., Bragstad K., Karlsson I., Krog J.S., Williams J.A., Fomsgaard A. (2015). Vector Optimization and Needle-Free Intradermal Application of a Broadly Protective Polyvalent Influenza A DNA Vaccine for Pigs and Humans. Hum. Vaccines Immunother..

[94] Barnes A.G., Barnfield C., Brew R., Klavinskis L.S. (2000). Recent Developments in Mucosal Delivery of PDNA Vaccines. Curr. Opin. Mol. Ther..

[95] Hobson P., Barnfield C., Barnes A., Klavinskis L.S. (2003). Mucosal Immunization with DNA Vaccines. Methods San Diego Calif.

[96] Jiang W., Ren L., Jin N. (2007). HIV-1 DNA Vaccine Efficacy Is Enhanced by Coadministration with Plasmid Encoding IFN-Alpha. J. Virol. Methods.

[97] Boyer J.D., Robinson T.M., Kutzler M.A., Vansant G., Hokey D.A., Kumar S., Parkinson R., Wu L., Sidhu M.K., Pavlakis G.N. (2007). Protection against Simian/Human Immunodeficiency Virus (SHIV) 89.6P in Macaques after Coimmunization with SHIV Antigen and IL-15 Plasmid. Proc. Natl. Acad. Sci. USA.

[98] Kalams S.A., Parker S.D., Elizaga M., Metch B., Edupuganti S., Hural J., De Rosa S., Carter D.K., Rybczyk K., Frank I. (2013). Safety and Comparative Immunogenicity of an HIV-1 DNA Vaccine in Combination with Plasmid Interleukin 12 and Impact of Intramuscular Electroporation for Delivery. J. Infect. Dis..

[99] Van Drunen Littel-van den Hurk S., Loehr B.I., Babiuk L.A. (2001). Immunization of Livestock with DNA Vaccines: Current Studies and Future Prospects. Vaccine.

[100] den Hurk V.D.L., Braun R.P., Lewis P.J., Karvonen B.C., Baca-Estrada M.E., Snider M., McCartney D., Watts T., Babiuk L.A. (1998). Intradermal Immunization with a Bovine Herpesvirus-1 DNA Vaccine Induces Protective Immunity in Cattle. J. Gen. Virol..

[101] Zheng C., Babiuk L.A., van Drunen Littel-van den Hurk S. (2005). Bovine Herpesvirus 1 VP22 Enhances the Efficacy of a DNA Vaccine in Cattle. J. Virol..

[102] Martin J.E., Sullivan N.J., Enama M.E., Gordon I.J., Roederer M., Koup R.A., Bailer R.T., Chakrabarti B.K., Bailey M.A., Gomez P.L. (2006). A DNA Vaccine for Ebola Virus Is Safe and Immunogenic in a Phase I Clinical Trial. Clin. Vaccine Immunol..

[103] Tebas P., Roberts C.C., Muthumani K., Reuschel E.L., Kudchodkar S.B., Zaidi F.I., White S., Khan A.S., Racine T., Choi H. (2017). Safety and Immunogenicity of an Anti-Zika Virus DNA Vaccine—Preliminary Report. N. Engl. J. Med..

[104] Henao-Restrepo A.M., Camacho A., Longini I.M., Watson C.H., Edmunds W.J., Egger M., Carroll M.W., Dean N.E., Diatta I., Doumbia M. (2017). Efficacy and Effectiveness of an RVSV-Vectored Vaccine in Preventing Ebola Virus Disease: Final Results from the Guinea Ring Vaccination, Open-Label, Cluster-Randomised Trial (Ebola Ça Suffit!). Lancet.

[105] Liu M.A. (2010). Immunologic Basis of Vaccine Vectors. Immunity.

[106] Corbett K.S., Edwards D.K., Leist S.R., Abiona O.M., Boyoglu-Barnum S., Gillespie R.A., Himansu S., Schäfer A., Ziwawo C.T., DiPiazza A.T. (2020). SARS-CoV-2 MRNA Vaccine Design Enabled by Prototype Pathogen Preparedness. Nature.

[107] Stitz L., Vogel A., Schnee M., Voss D., Rauch S., Mutzke T., Ketterer T., Kramps T., Petsch B. (2017). A Thermostable Messenger RNA Based Vaccine against Rabies. PLoS Negl. Trop. Dis..

[108] Guidelines for Assuring the Quality, Safety, and Efficacy of Plasmid DNA Vaccines—Post ECBS Version/1 September 2020. https://www.who.int/publications/m/item/DNA-post-ECBS-1-sept-2020.

